# Beyond the Pill Bottle: Time to follow the treatment evidence for Borderline Personality Disorder

**DOI:** 10.7748/mhp.2025.e1728

**Published:** 2025-01-28

**Authors:** D Whittaker, S Lamont, J Harrison, S Richardson, K Berzins

**Affiliations:** Lancashire and South Cumbria NHS Foundation Trust; University of Lancashire; Lancashire and South Cumbria NHS Foundation Trust; University of Lancashire

## Abstract

Borderline personality disorder is arguably the most prevalent personality disorder seen in healthcare settings, characterised by emotional instability, impulsive behaviours, distorted thinking, and unstable relationships. This commentary explores the effectiveness of pharmacological treatments for borderline personality disorder, drawing on the findings from a Cochrane review. It highlights the limited evidence supporting the efficacy of psychotropic medications such as antipsychotics, antidepressants, or mood stabilizers in improving BPD symptoms, self-harm, suicidal behaviours, or psychosocial functioning. Despite limited evidence, many people with borderline personality disorder receive psychotropic medications, often long-term and with risks of polypharmacy. A cautious approach to psychotropic medication is needed in addition to improvements in research rigor for pharmacotherapy trials. The limited evidence urges judicious prescribing, addressing stigma in practice, and prioritising non-drug options. Non-pharmacological psychotherapies have more support, yet access barriers persist. Monitoring and deprescribing plans are also recommended, as is research examining prescriber behaviour and integration of persons with lived experience.

## Background

Borderline personality disorder (BPD) is categorized as a personality disorder and is distinguished by at least five or more of the following criteria: avoiding abandonment, unstable and intense relationships, identity disturbance, impulsivity, recurrent suicidal or self-harming behaviour, affective instability, chronic feelings of emptiness, anger and transient, stress-related paranoid ideation (APA 2022). BPD causes significant impairment and distress to those who are affected and is associated with multiple medical and psychiatric co-morbidities (Tomko et al 2014, Chapman et al 2023), and greater use of medical services (Leichsenring et al 2023). Prevalence of BPD is suggested to be roughly 1% in community populations, 10-12% in outpatient psychiatric settings and 20-22% among inpatient settings (Ellison et al 2018). Prevalence rates are slightly higher in females, people in lower income brackets, people younger than 30 and people who are separated or divorced (Tomko et al 2014).

Recent updates in the Diagnostical and Statistical Manual of Mental Disorders, Fifth Edition (DSM-5) and International Classification of Diseases 11^th^ Revision (ICD-11) have introduced more dimensional approaches to personality disorders. The DSM-5 includes an alternative model focusing on personality traits and severity, while the ICD-11 emphasises trait domains and a borderline pattern (APA 2022, WHO 2019). These changes reflect a shift towards a more nuanced and individualised understanding of personality disorders, which may influence the diagnosis and treatment of borderline personality disorder.

In the US, the American Psychiatric Association Guideline (APA 2001) recommends psychotherapy as the primary treatment for patients with BPD, and an updated guideline remains in draft form. In Europe and the UK, guidance does not recommend the role of drug treatment for BPD specifically but advises that it may be considered in the overall treatment of comorbid conditions (NICE 2009, Simonsen et al 2019). Despite this, UK data from a cross-sectional survey of self-selected psychiatric services reported that in a sample of people diagnosed as having emotionally unstable personality disorder (n=1776), 92% were prescribed psychotropic medication, likely to be an antidepressant or antipsychotic, and prescribed primarily for symptoms and behaviours of the condition, particularly affective dysregulation (Paton et al 2015). Additionally, a recent nationwide study of patients in the UK within primary care settings found that a quarter of people with any recorded personality disorder, who did not also have a major mental illness, were prescribed antipsychotics (Hardoon et al 2022). Of the same group, 18% were prescribed antipsychotics for more than a year and 11% for more than five years. (Hardoon et al 2022).

Given the use of pharmacological intervention for the treatment of BPD, it is critical to consider the evidence-base. A Cochrane systematic review from 2010 suggested that drug treatment, especially mood stabilisers and second-generation antipsychotics may have a role to play in addressing core symptoms and related psychopathology, but the evidence did not support their effectiveness in reducing overall BPD severity (Lieb et al 2010). Further systematic reviews concluded that whilst some pharmacological treatments showed some promise in targeting specific BPD symptoms, there was a dearth of high-quality evidence to make informed decisions regarding the use of pharmacology for the treatment of BPD (Gartlehner et al 2021, Hancock-Johnson et al 2017). In 2022, an update of the Cochrane review on pharmacological interventions in the treatment of BPD sought to explore the more recent evidence with a more comprehensive search strategy (Stoffers-Winterling et al 2022). This critical commentary will discuss the findings of the systematic review, critically appraise the methods used and expand upon the findings in the context of clinical practice and further research.

## Methods of the review by Stoffers-Winterling et al (2022)

A comprehensive search of multiple databases including CENTRAL, MEDLINE and Embase was undertaken up to February 2022. Hand searches of trial registries, relevant journals, unpublished data and cross references were also completed. As the review was one of a wider series of interventions for BPD, the search strategy included all psychotherapeutic or pharmacological treatment of BPD. Studies were included if they were randomised controlled trials (RCTs) of any medication to treat the disorder or symptoms (including a combination of medications where defined), and at any dosage (where the prescription was given continuously for at least 2 weeks). Comparators were placebo or active comparator medications. Miscellaneous medications included omega-3 fatty acids, which the authors imply were tested due to potential mood stabilising effects (Stoffers et al. 2010). Trials with a supplementary intervention such as psychological therapies were included if the treatment group were the unique recipients of the pharmacological intervention. Screening for included studies was undertaken by 13 reviewers working independently in pairs, with disagreements resolved by consensus or third author. All authors extracted data independently on standardised data extraction forms with disagreements resolved by discussion or an arbiter if required. Quality assessment was undertaken by all review authors, and by means of the Cochrane collaboration’s tool for assessment of risk of bias. Trials were categorised as low or high risk of bias overall. Outcome measures could be self-rated or clinician-rated using validated measures. Primary outcomes were BPD severity, the proportion of patients with self-harming behaviour, psychosocial functioning and suicide related outcomes. Secondary outcomes comprised of depression, anger and adverse events. Statistical heterogeneity of included trials and subgroup/sensitivity analyses were undertaken. Where possible, data from primary trials were pooled in a meta-analysis and reported with 95% CIs. Where it was inappropriate to do this due to high levels of heterogeneity, a narrative description of results was provided. The GRADE approach was used by two authors independently to rate the certainty of evidence.

## Results of Stoffers-Winterling et al (2022)

From 28,486 records, 907 full texts were screened and 46 RCTs were included with 45 trials and 2752 participants eligible for quantitative analysis. Mean age of participants ranged from 16.2-39.7 years and most trials included both sexes although predominantly female. Fifteen trials were female only and one male only. Thirty-two trials took place in an outpatient setting, 9 an in-patient setting, 4 a combination of both and 1 unstated. Twenty trials were from Europe, 20 from the US, 3 from South-West Asia, 1 from Australia and 2 multi-country. All trials were assessed as having a high risk of bias.

### BPD Symptom Severity

The evidence of effect for anti-psychotic medication suggested little to no difference on BPD symptom severity when compared with placebo, based on very low certainty evidence (SMD-0.18, 95% CI-0.45 to 0.08; p=0.18). Anti-depressants compared to placebo post-treatment indicated little to no difference based on very low certainty evidence (SMD 0.27, 95% CI −0.65 to 1.18; p=0.57) as did mood stabilisers (SMD 0.07, 95% CI −0.43 to 0.57; p=0.78). There was no evidence of effect for miscellaneous medications.

### Self-harm

The evidence of effect for antipsychotics (RR 0.66, 95% CI 0.15 to 2.84; p=0.57), antidepressants (MD 0.45, 95% CI −10.55 to 11.45; p=0.94) and mood stabilisers (RR 1.08, 95% CI 0.79 to 1.48; p= 0.64) on self-harm to placebo at the end of treatment was very uncertain and based on one or two trials and very low certainty evidence. There was no clear evidence of effect for Omega-3 on self-harm.

### Suicide-related outcomes

The evidence of effect for anti-psychotics (SMD 0.05, 95% CI -0.18 to 0.29; p=0.67), antidepressants (SMD −0.26, 95% CI −1.62 to 1.09; p=0.70); and mood stabilisers (SMD -0.36, 95% CI -1.96 to 1.25; p=0.66) to placebo suggested little to no effect on suicide related outcomes at the end of treatment based on very low certainty evidence. In relation to miscellaneous medications, the benzodiazepine alprazolam showed no clear evidence of difference to placebo, however omega-3 may reduce suicide related outcomes at the end of treatment (RR 0.52, 95% CI 0.28 to 0.95; p=0.03) but this was based on one trial only.

### Psychosocial functioning

The evidence of effect for anti-psychotics (SMD −0.16, 95% CI −0.33 to 0.00; p=0.05), antidepressants (SMD -0.25, 95% CI −0.57 to 0.06; p=0.11, and mood stabilisers (SMD −0.01, 95% CI −0.28 to 0.26; p=0.94), compared to placebo suggested little to no difference in psychosocial functioning at the end of treatment based on very low certainty evidence. There was evidence of a difference for Omega-3 fatty acids but this was based on one trial only.

### Secondary outcomes and adverse events

Evidence of effect for secondary outcomes including anger, affective instability, feelings of emptiness, impulsivity, abandonment, identity disturbance, dissociation and psychotic-like symptoms, depression and attrition was inconclusive. Anti-psychotics and mood stabilisers may slightly lessen interpersonal problems based on low certainty evidence. Very low certainty and limited data were obtainable for serious adverse events.

### Sub-group Analyses

Sub-group analyses were undertaken for medication type, substances, level of psychosocial functioning, setting, funding, and trial size. For the outcome of BPD severity, It was suggested that first-generation anti-psychotics showed inferiority compared to second generation anti-psychotics. For BPD severity, there was also a higher intervention effect of those trials which received funding from the pharmaceutical industry.

## Commentary

The AMSTAR2 critical appraisal tool (Shea et al 2017) was employed to assess the methodological quality of the review by Stoffers-Winterling et al 2022. Of the 16 AMSTAR 2 criteria, 15 were met (see Table 1.), indicative of a robust and comprehensive summary of evidence. The only criterion that was not met related to the justification for including RCTs only, with no explanation as to why observational studies were not included. Overall, the review scored high and provides an accurate and comprehensive summary of the results of the available studies that address the question of interest.

Stoffers-Winterling et al (2022) identified that interventions using antipsychotics, antidepressants and mood stabilisers had little evidence of effect on BPD symptom severity, self-harm, suicidal behaviours and psycho-social functioning for people with BPD based on mostly very low certainty evidence. No miscellaneous medications included in the trial had any effect on outcomes compared to placebo, apart from omega-3 fatty acids which may influence psychosocial functioning and suicide related outcomes. Secondary outcomes were also inconclusive and limited data were obtainable for adverse events. Despite additional data from 18 further studies, the conclusions from this updated Cochrane review remain the same as the 2010 version; there are likely no benefits of medication for BPD but the evidence remains uncertain.

UK guidance states that drug treatments should not be used specifically for BPD or for the individual symptoms or associated behaviour (NICE 2009). Furthermore, guidance on managing a crisis in BPD advises that a drug treatment is not used in place of other more appropriate interventions, polypharmacy should be avoided and a single drug used, with a plan to stop drug treatment within 1 week and regular review of the drug treatment if this is not possible (NICE 2009). In July 2018, following the revision of the International Classification of Diseases (ICD-11), NICE reviewed the 2009 guidance and found no new evidence that affected their recommendations, confirming their continued relevance. Despite this guidance, and a lack of evidence to support prescribing for BPD, psychotropic medication prescribing is common in patients with personality disorder and is often prescribed for prolonged periods of time (Zanari et al 2015, Kadra-Scalzo et al 2021, Hardoon et al 2022). Indeed, pharmacotherapy is substantially more common than recommended in clinical guidelines despite no drugs having been specifically approved for treatment of BPD including in America and some European countries (Pascual et al 2023). Polypharmacy amongst in-patients is also common (Bridler et al 2015) and post-discharge deprescribing is not widely practiced (Shapiro-Thompson and Fineberg 2022). This is not without risk, as increased polypharmacy in BPD is recognised to increase the possibility of adverse drug reactions, medication interactions and cumulative toxicity (Kukreja et al 2013) as well as detrimental health outcomes, such as readmission into hospital (Kadra et al 2018). Antipsychotic medication and to a more restricted degree antidepressants and mood stabilizers, are also associated with an increased risk of cardiometabolic side effects as well as movement and seizure disorders (Correll et al 2015).

People with BPD who are prescribed pharmacological treatment may also be exposed to inequalities of treatment. There is a lack of recommended monitoring of anti-psychotic medication in this population, who do not fit within the definition of Serious Mental Illness (SMI) and subsequent physical health monitoring protocols (Hardoon et al 2022). Furthermore, it has been reported that patients with BPD are viewed with a negative attitude by mental health professionals (Papathanasiou and Stylianidis 2022) and are commonly described in terms such as ‘attention-seeking’, ‘difficult’, and ‘manipulative’ (Masland et al 2023). This can have a significant influence on treatment for those with BPD as clinicians across disciplines commonly express less sympathy, empathy and optimism, and more hostility toward patients with BPD (Masland et al 2023). The overlap of BPD symptoms with other mental health diagnoses symptoms, may also result in misdiagnosis and unsuitable treatments such as overuse of pharmacy rather than psychotherapy (Ociskova et al 2023). BPD patients also experience greater levels of deprivation relative to the general population and individuals with BPD living in areas with greater deprivation are more likely to be prescribed antipsychotics (Hardoon et al 2022).

Given the lack of evidence for pharmacotherapy and complications of prescription within the BPD population, it is important to highlight the non-pharmacological treatment interventions for BPD. Recent research reflects this growing emphasis on non-pharmacological treatments. Over the past two decades, psychological therapies such as Dialectical Behaviour Therapy (DBT), Cognitive Behavioural Therapy (CBT), Mentalization-Based Therapy (MBT), and Schema Therapy (Bateman & Fonagy 2006, Linehan 1993, Young et al. 2003), have been prioritised due to their proposed effectiveness in managing core symptoms and improving emotional regulation and interpersonal functioning (Liu et al 2024).

A Cochrane systematic review concluded that there is reasonable, although low quality evidence, that psychotherapeutic interventions are helpful for people with BPD (Storebo et al 2020). Other reviews have indicated similar effects but with low levels of evidence certainty (Cristea et al 2017, Oud et al 2018, Spong et al 2021, Setkowski et al 2023). In the UK, guidance advises that when providing psychotherapy sessions for people with BPD, an explicit and integrated theoretical approach should be used which is adapted to the person’s need and context of living, and for no less than 3 months’ duration with twice weekly sessions considered (NICE 2009). Between thirteen to eighteen sessions of psychotherapy may also be needed for 50% of patients to aid recovery (Hansen et al 2006).

Long term psychological therapies however have limited availability (Paris 2013), and variation exists for which specialist mental health services are available in the UK, leading to a ‘postcode lottery’ (NCCMH 2019). For many people with severe BPD, they are also unable to engage or drop out of treatment before completion (Crawford et al 2009, McMurran et al 2010, Dixon and Linardon 2020) with the highest dropouts in the first quarter of treatment and for those treatments provided as a group rather than on an individual basis (Arntz et al 2022). Furthermore, Woodbridge et al (2022) suggest that approximately half the people who receive psychotherapy for borderline personality disorder do not respond to treatment regardless of treatment type or treatment length, with contributing factors to non-response unclear. Early diagnosis, and the right treatment and dose of psychotherapy could avoid lengthy and expensive treatment, improving longer term outcomes and best benefits (Campbell et al 2021). The provision of psychotherapy such as DBT may also have a positive effect on reducing healthcare utilisation and related health care costs, particularly for in-patient admissions and emergency contacts (O’Sullivan et al 2017) and can be considered cost-effective in the short-term (Murphy et al 2020).

## Conclusion

In conclusion, addressing the use of psychotropic medications in BPD requires a more robust evidence base, emphasizing research rigor, diversity in study populations, and substantial sample sizes. Current evidence urges caution in initiating psychotropic medications, advocating a case-by-case approach for acute exacerbations or crises, with full patient information and regular regimen reviews. For people with BPB, clear communication from mental health practitioners on the uncertain outcomes for pharmacological treatments is required, in addition to up-to-date evidence for non-pharmacological treatments. A deeper understanding of BPD’s pathophysiology could also help reduce stigma and enhance medication development. Prescribers should carefully consider outcome measures, and future research should prioritize placebo-controlled trials alongside evidence-based talking therapies. Additionally, research should explore prescriber behaviour and literacy and include individuals with lived experience in the research process. Antipsychotic monitoring should be a standard practice, irrespective of diagnosis.

### Relevance for clinical practice and further research

- Pharmacological interventions had little evidence of effect on BPD severity, self-harm, suicidal behaviours and psycho-social functioning for people with BPD based on evidence of very low quality.
- The uncertain evidence suggests caution in initiating psychotropic medications in BPD.
- There is a need for more robust evidence commensurate with the level of psychotropic prescribing in BPD.
- Greater rigor in research methodology, which is representative of diverse demographic cohorts, minimises bias, and has significant sample power is warranted.

### CPD reflective questions

- Why is BPD such a stigmatised diagnosis?
- What treatments and interventions for BPD are supported by evidence?
- Why are psychotropic medications prescribed so frequently to people with BPD?

## Figures and Tables

**Table 1:** Critical appraisal using the AMSTAR-2 tool for assessing systematic reviews

AMSTAR-2 items	Responses
1. Did the research questions and inclusion criteria for the review include the components of PICO?	Yes: all components of the PICO were met.
2. Did the report of the review contain an explicit statement that the review methods were established prior to the conduct of the review and did the report justify any significant deviations from the protocol?	Yes: a new protocol was developed and published prior to conducting the review and methods that were unable to be used were reported.
3. Did the review authors explain their selection of the study designs for inclusion in the review?	No: rationale not provided for inclusion of RCT only.
4. Did the review authors use a comprehensive literature search strategy?	Yes: a comprehensive multi-database search was undertaken up to Feb 2022 in addition to hand searching of trial registries.
5. Did the review authors perform the study selection in duplicate?	Yes: reviewers worked in pairs and independently screened with consultation by a third reviewer if required.
6. Did the review authors perform data extraction in duplicate?	Yes: review authors worked in pairs and completed data collection forms independently with disagreements resolved by discussion or arbitration.
7. Did the review authors provide a list of excluded studies and justify the exclusions?	Yes: potentially relevant RCTs that did not fulfil the inclusion criteria were reported with reasons for exclusion.
8. Did the review authors describe the included studies in adequate details?	Yes: characteristics of included studies were reported.
9. Did the review authors use a satisfactory technique for assessing the risk of bias in the individual studies that were included in the review?	Yes: Cochrane’s risk of bias tool.
10. Did the review authors report on the sources of funding for the studies included in the review?	Yes: funding sources were reported.
11. If meta-analysis was performed did the review authors use appropriate methods for statistical combination of results?	Yes: statistical analyses were reported according to Cochrane Handbook recommendations.
12. If meta-analysis was performed did the review authors assess the potential impact of RoB in individual studies on the results of the meta-analysis or other evidence synthesis?	Yes: the GRADE approach considered risk of bias when grading certainty of evidence.
13. Did the review authors account for RoB in individual studies when interpreting/discussing the results of the review?	Yes: evidence was graded as very uncertain and discussed accordingly.
14. Did the review authors provide a satisfactory explanation for and discussion of, any heterogeneity observed in the results of the review?	Yes: clinical, methodological and statistical heterogeneity were assessed, and relevant sub and sensitivity-analyses were performed.
15. If they performed quantitative synthesis did the review authors carry out an adequate investigation of publication bias and discuss its likely impact on the results of the review?	Yes: inspection of funnel plots and Egger’s tests were undertaken. Publication bias was considered within the GRADE approach.
16. Did the review authors report any potential sources of conflict of interest, including any funding they received for conducting the review?	Yes: declarations of interest were provided
